# 

**Published:** 1881-11-20

**Authors:** 


### EDITORIALS AND MISCELLANEOUS.

☞ READ !—All subscribers who do not notify us to discontinue by the 10th of January next will be entered upon the list for 1882.

We trust that no one will stop his Journal, but that all will pay up their subscriptions, and start us on a new year with glad hearts, with our hopes brightened and our energies stimulated for more vigorous and successful work in the future.

We submit to our subscribers this

LIBERAL PROPOSITION :

Every subscriber who will send us three new names and six dollars can get the Journal for 1882 free of expense.

R. C. WORD, M. D., Managing Editor.

Receipts of subscribers will appear in the next issue.

